# PBL-based online-offline hybrid teaching in nursing education: enhancing self-regulated learning and teaching effectiveness in China

**DOI:** 10.3389/fmed.2025.1601003

**Published:** 2025-11-26

**Authors:** Xiaoyan Feng, Ying Zhou, Bizhen Liao

**Affiliations:** 1 Department of Obstetrics and Gynecology, The First Affiliated Hospital of Chongqing Medical University, Chongqing, China; 2 Department of Nursing, The First Affiliated Hospital of Chongqing Medical University, Chongqing, China

**Keywords:** PBL, online-offline, nursing education, self-regulated learning, teaching effectiveness

## Abstract

**Objective:**

This study aimed to evaluate students’ satisfaction and learning outcomes associated with a blended online and offline teaching method based on problem-based learning (PBL).

**Design:**

A total of 238 third-year undergraduate nursing students from two classes participated. Each class was divided into eight groups, with 14 to 15 students per group. Eight teachers were involved. A cross-sectional survey was conducted among students after class.

**Methods:**

Hypertensive disorders of pregnancy were chosen as the teaching cases. Teachers uploaded teaching resources to Superstar Learning App for 1 week of online self-regulated learning (SRL). During the offline class, each group addressed two clinical questions through presentations, simulated scenarios and role-playing exercises. After course, students were invited to complete an anonymous satisfaction questionnaire. A descriptive cross-sectional design was employed, a descriptive analysis of participants was conducted, and factors influencing teaching effectiveness were analyzed.

**Results:**

Overall, 79.82% of students expressed greater satisfaction with this blended method compared to traditional teaching approaches, 95.07% of students gave positive feedback on the course, and 92.82% reported that their learning outcomes either met or exceeded expectations. Students who engaged intensively in collecting, analyzing, and organizing information during the online phase, or who devoted ≥5 h to online learning, were significantly more likely to achieve or surpass their learning expectations (*p* < 0.05). Although participation in activities did not significantly affect learning outcomes (*p* > 0.05), it did influence students’ evaluation of teaching (*p* < 0.05). A significant difference was also observed between the two classes in their session evaluations (*p* < 0.05).

**Conclusion:**

The blended PBL approach was associated with higher student satisfaction in the Obstetrics and Gynecology Nursing course. Class participation correlated positively with evaluations and outcomes. While active participants achieved expected results, less participative students also attained comparable outcomes through alternative methods. Although pre-class preparation time predicted learning effectiveness, efficiency was equally significant. Adopting a student-centered, PBL-oriented approach with diversified resources is recommended to address individual needs, enhance satisfaction, and develop SRL skills. This model promotes SRL by encouraging students to assess and adjust their learning strategies, thereby meeting diverse needs and supporting personalized development.

## 1 Introduction

Problem-based learning (PBL) is a student-centered, teacher-guided teaching method centered on problem-solving (1). In the early 1990s, research in this field primarily focused on critical analyses of PBL teaching effectiveness. Since the turn of the 21st century, emphasis has shifted toward comparative studies on curriculum design (2–4). Recent research in China has explored the integrated application of PBL with other pedagogical approaches and their synergistic effects (5, 6). PBL was introduced into Obstetrics and Gynecology in the early 21st century (7) and subsequently applied to Obstetric Nursing education (8). Along with traditional lectures, heuristic discussions, case-based teaching, learning tools, and multimedia instruction, PBL has become one of the core methods used in Obstetrics and Gynecology Nursing education (9, 10). Compared to traditional Lecture-Based Learning (LBL), PBL has demonstrated greater effectiveness in surgical education by enhancing clinical competence, student satisfaction, problem-solving skills, SRL, and critical thinking abilities (11, 12). Recently, hybrid PBL models have gained popularity, such as PBL combined with mind mapping in nursing education to improve self-learning (13), and PBL integrated with Case-Based Learning (CBL) to enhance performance and clinical skills among medical students and residents (14).

The current Obstetrics and Gynecology Nursing course has been recognized as a national-level first-class undergraduate course featuring online and offline hybrid instruction. The chapter “Nursing Care for Women with Hypertensive Disorders of Pregnancy” is well-suited for PBL due to its practice-oriented nature. PBL emphasizes pre-class SRL and teamwork to prepare students for in-class discussions (15).

This study employed a blended online-offline teaching model based on PBL to explore its application in theory and practice teaching aimed at developing nursing competence in managing Hypertensive Disorders of Pregnancy. It also investigated factors influencing teaching effectiveness, such as student satisfaction, adequacy of pre-class preparation, and classroom participation. The online component focused on theoretical knowledge through SRL, where students acquired knowledge and completed task based questionnaires provided by the teacher who complemented by faculty support through resource provision, answering question and feedback. The offline component adopted PBL teaching method, activities including student presentations, discussions, instructor summaries, as well as hands-on operational exercises and drill observations. Both online and offline learning were problem-oriented, and the integration of content and format across both modalities facilitates efficient acquisition of theoretical knowledge and proficient mastery of clinical skills.

## 2 Methods

### 2.1 Participants

A total of 238 third-year undergraduate nursing students from two classes at Chongqing Medical University were enrolled as participants. Both classes followed the same curriculum and were taught by the same team of eight teachers. Each class was divided into eight groups, resulting in a total of 16 groups. Each group consisted of 14 or 15 members, assigned evenly according to student ID number. Each group was further subdivided by the student leader into four subunits. The teachers assigned four questions, with one subunit responsible for addressing one question. Each teacher was assigned to facilitate two student groups across the two classes. For instance, Teacher A conducted offline sessions for Group 1 of Class 1 on 1 day and for Group 1 of Class 2 on another day (Class 1 on April 15, 2024, Class 2 on April 16, 2024). All teachers were nurses with at least 5 years of clinical nursing experience and held university teaching qualifications. The course coordinator provided pre-course training on the PBL teaching model to all teachers.

One week before the offline session, online teaching activities were carried out via the Superstar Learning App. These activities combined SRL with teacher guidance and included post lesson exercises. Teaching evaluation tasks were uniformly administered by the teaching secretary. Online instruction was conducted simultaneously for both classes, with teachers responsible for answering questions and providing feedback to their assigned groups. Offline teaching was delivered in two separate classroom sessions, one for each class. The offline component adopted the PBL model, with pre-released questions. Student presentations served as the core activity, supplemented by teacher explanations and summaries. During the online preparation phase, each group was assigned specific tasks. In the offline speaking session, group representatives delivered presentations, and discussion sessions involved voluntary participation, determined by the number of times each student spoke.

### 2.2 Teaching methods

The teaching objectives of this course were to develop students’ SRL skills, enhance their critical thinking abilities, and strengthen their capacity to solve clinical problems. Simultaneously, the design aimed to boost students’ learning motivation and satisfaction with instruction.

The educational approach was student-centered, with teachers serving in a facilitative role. The instructional design was problem-oriented and integrated both online and offline modes to deliver teaching activities. No other parallel learning activities were conducted.

**Figure 1:**
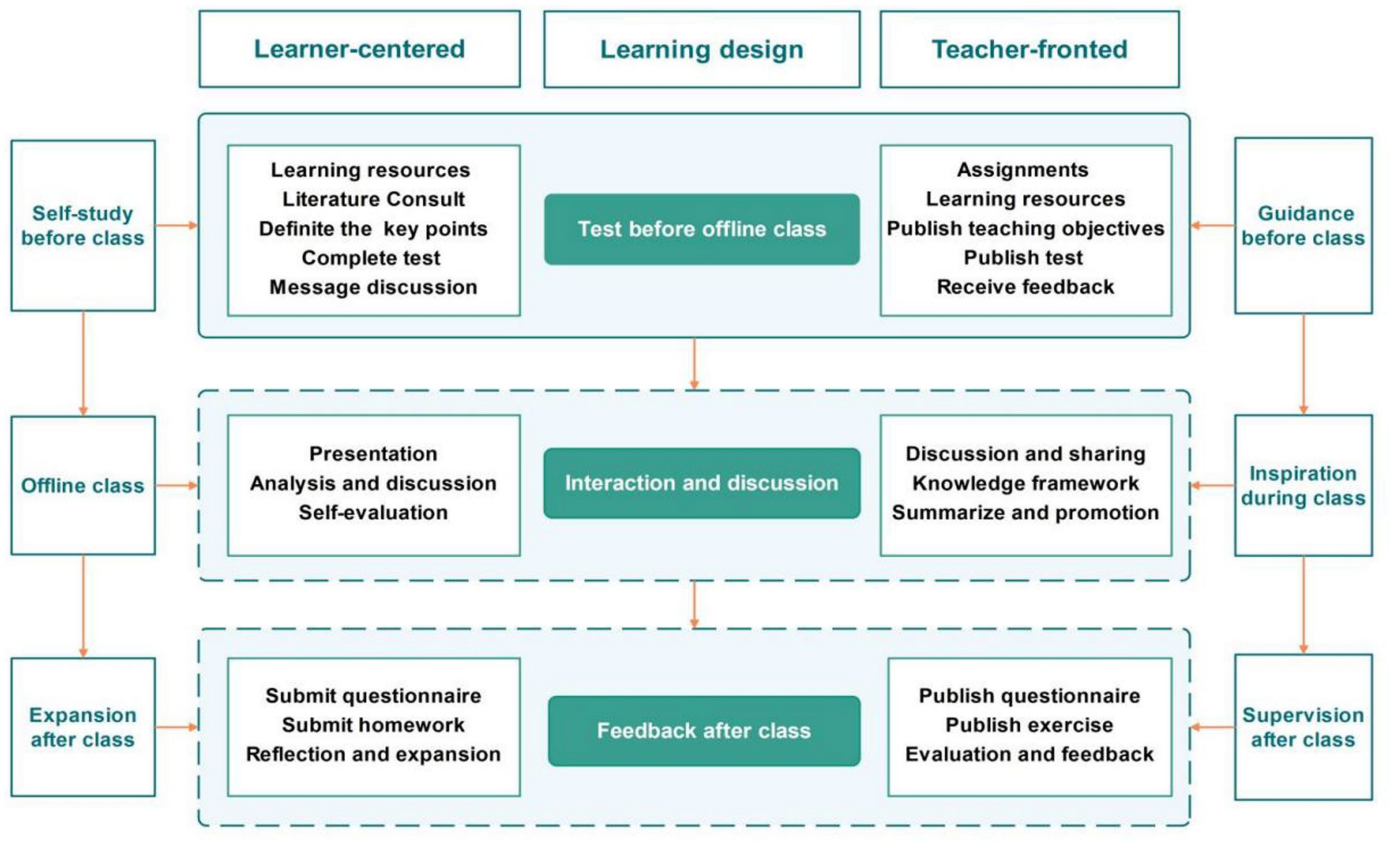
Course design.

The course content focused on the nursing care of women with gestational hypertension. Teaching activities were built around an authentic clinical case of gestational hypertension. Teachers posed questions which related to the case to guide students’ online self-study. During offline sessions, students presented their solutions, engaged in class discussions, and benefited from knowledge extension provided by the instructor. After class, exercises were assigned to reinforce learning, and teaching effectiveness was evaluated (see Figure 1).

#### 2.2.1 Self-study online

Prior to the class, the teachers prepared lesson materials covering both core knowledge and clinical practices to ensure adequate depth and breadth of the content. One week before the class, teaching resources including micro-lecture videos, presentation slides, recommended references, quizzes, and other learning materials were uploaded to the online platform to facilitate easy access and self-directed learning. The online learning period for Class 1 and Class 2 was scheduled from April 8 to April 14, 2024.

Simultaneously, the teachers announced the learning plan on the platform to help students clearly understand their learning tasks, which included clinical case studies, discussion questions, and specific requirements for group collaboration. The uploaded resources comprised various materials related to gestational hypertension, allowing students to selectively study and utilize them according to their needs. Additionally, students were required to independently search relevant literature and textbooks based on the assigned problems.

The online component was problem-oriented, encouraging students to design their own formats for responding to questions. This approach aimed to develop their ability to efficiently filter information, think critically, and apply knowledge through self-directed processes. Teachers monitored students’ online progress through the platform and adjusted the lesson plan accordingly 1 day before the offline session. During the online self-learning phase, teachers served as facilitators, making timely adjustments to the offline course schedule, supplementing key points, and modifying teaching difficulties based on students’ feedback. Students engaged in self-paced online learning, which involved literature review, case analysis, and group discussions. They prepared presentation slides according to their study plans and got ready for offline presentations and discussions. An online quiz was completed 2 days before the offline class. Throughout the week, students could seek help from teachers via the platform.

Adopting a student-centered approach, problem solving tasks were led by the students with support from the teachers. Students had the autonomy to decide how to address the problems, whether by creating PowerPoint presentations (using both provided resources and self sourced materials) or through scenario-based exercises such as scriptwriting and role-playing to demonstrate knowledge and skills. The online courses were accessible without time restrictions, provided that all tasks were completed before the offline session.

#### 2.2.2 Offline study

The offline course was structured around a three act case scenario and eight key issues. It consisted of two class sessions, totaling 80 min (Class 1 on April 15, 2024, and Class 2 on April 16, 2024). The content covered risk factors, etiology, clinical manifestations, and classification of Hypertensive Disorders of Pregnancy, as well as nursing diagnoses, admission procedures, medication management, and emergency protocols for eclampsia.

Specifically, both online and offline sessions were centered on an authentic clinical case, which included four guiding questions (see Table 1). Students were encouraged to think critically, discuss, and make clinical decisions. Teachers helped students organize knowledge frameworks and integrate interdisciplinary content from internal medicine, pharmacology, emergency nursing, psychology, and sociology into Obstetrics and Gynecology Nursing.

**Table 1:** PBL problem list and student answering methods.

Questions	Presentation format	Problem solving methods
Question 1: Analyze the causes of Ms. Wang’s illness and list the high-risk factors for her illness.	PowerPoint presentation explanation	Explain theoretical knowledge points and analyze case studies (pathogenic factors)
Question 2: Identify the diagnostic basis for Ms. Wang’s severe preeclampsia?	PowerPoint presentation explanation	Analyze case
Question 3: As a receiving nurse, how would you provide initial care for Ms. Wang, who has just been admitted to the hospital?	Role-playing	Present nursing procedures and apply nursing measures through case studies
Question 4: Based on the examination and testing results, supplement Ms. Wang’s medical diagnosis and evidence after admission?	PowerPoint presentation explanation	Analyze case

In each class, students were divided into four groups, with each group assigned two questions to address. Groups shared their responses through presentations, scenario simulations, and role-playing exercises. Following each presentation, an open discussion was held where group members supplemented each other’s answers, and students raised questions and exchanged opinions. Finally, the teacher summarized the session, consolidated the knowledge framework, and assigned homework.

#### 2.2.3 Feedback and development

Teaching evaluation was conducted through a questionnaire survey that assessed students’ mastery of key knowledge points, satisfaction with the course, and level of engagement in classroom activities. The teachers provided a Quick Response code for the teaching effectiveness questionnaire, and all students completed it within 1 h after class. Informed consent was obtained from the students, and the personal information collected in the survey questionnaires was explicitly promised to be kept confidential. The Teaching Satisfaction Survey was developed internally based on the course content and students’ post class assessment performance, and it was reviewed by experts from the course development team before implementation.

The post course test consisted of five questions based on the classroom case, covering the diagnosis, pathological changes, causes, nursing measures, and health related issues of gestational hypertension. The questions included both single choice and multiple choice formats. The accuracy rate calculated from the post test responses was used to evaluate students’ knowledge acquisition.

The teaching evaluation included self assessment, peer evaluation, and teacher assessment. The questionnaire addressed students’ participation, perceived teaching effectiveness, and overall course evaluation. Classroom participation was evaluated from three aspects:active involvement in discussions (measured by the number of voluntary speeches, as recorded by the teacher), enthusiasm in completing tasks, and contribution to collaborative activities. Both student self rating and teacher evaluation were employed. Students self assessed based on their own perceived performance, while instructors quantified students’ active participation during discussions and rated their task engagement, including pre-class preparation, problem reporting, and online activity regarding post-class questions.

Additionally, the teacher assigned exercises and extended discussion topics, such as, what are the latest research advances in the etiology and pathology of Hypertensive Disorders in Pregnancy? Students completed these assignments within 1 week after the course through literature review.

### 2.3 Statistical analysis

Statistical analysis was performed using SPSS version 26.0. Descriptive data were presented as counts and rates. Group comparisons were conducted using the *χ*^2^ test, with a significance level set at *p* < 0.05, and calculated the effect size. This study is descriptive in nature, all participants were surveyed without predefined control or intervention groups. Differences were explored through univariate analysis with artificially categorized influencing factors.

## 3 Results

### 3.1 Sociodemographic information

The total number of students was 238, female (96.6%) and eight were male (3.4%), all of whom were undergraduate students majoring in nursing. The average age was (21.00 ± 0.66) years old.

### 3.2 The modified blended PBL education evaluation

A total of 238 students participated in the online and offline PBL courses. After excluding incomplete questionnaires and those completed in less than 1 min (as a pre-test indicated that careful completion required at least 1 min, responses submitted more quickly were considered potentially random and thus excluded to maintain objectivity in teaching evaluation), 223 valid questionnaires were included in the final analysis, 118 were from Class 1 and 105 from Class 2.

**Figure 2:**
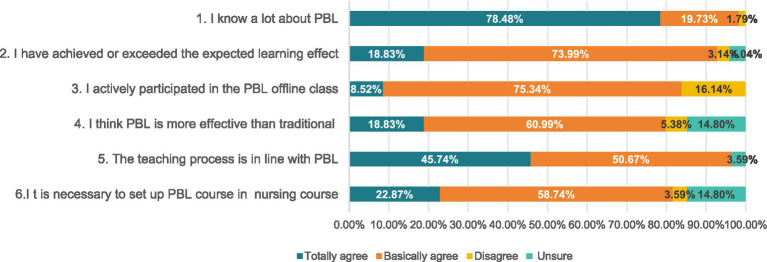
Evaluation of modified blended PBL application in Obstetrics and Gynecology Nursing education(%).

Analysis revealed that 78.48% of the students were highly familiar with the PBL approach. Additionally, 92.82% of respondents (18.83% strongly agreeing, 73.99% somewhat agreeing) believed that the course met their learning expectations. Although 16.14% of students reported not actively participating during offline sessions, 79.82% (18.83% strongly agreeing, 60.99% somewhat agreeing) indicated that the PBL teaching method was more effective than traditional instruction. Furthermore, 96.41% (45.74% strongly agreeing, 50.67% somewhat agreeing) agreed that the course implementation adhered to standard PBL procedures, and 82.61% (22.87% strongly agreeing, 58.74% somewhat agreeing) expressed that offering a PBL course on Hypertensive Disorders of Pregnancy was beneficial (see Figure 2).

Among the six questions evaluating the quality of PBL teaching, the “unsure” option accounted for a certain proportion in four of them. For the statements “I think PBL is more effective than traditional teaching methods” and “It is necessary to set up PBL courses in nursing programs,” the proportion of “unsure” responses was 14.80% for both.

### 3.3 The effect of students’ class participation for teaching evaluation

Among students who reported being “highly active” in class, 89.47% rated the course as “excellent,” whereas only 50.00% of less active participants gave an “excellent” rating. Increased participation was significantly associated with higher course evaluations (*p* < 0.05, |*φ*| = 0.309). The analysis also revealed that 59.32% of students in Class 1 and 78.10% in Class 2 rated the course as “excellent.” A significant difference in overall course evaluation was observed between the two classes (*p* < 0.05, |*φ*| = 0.203, see Table 2).

**Table 2:** Analysis of the relationship between student class participation and teaching evaluation (%).

Influence factors	Cases	Evaluation level				χ2	*p*	*|φ|*
		excellent	good	medium	poor			
Participation enthusiasm (self-evaluation)						21.32	0.002	0.309
Very enthusiastic	19 (8.52)	17 (89.47)	2 (10.53)	0 (0.00)	0 (0.00)			
Enthusiastic	71 (31.84)	55 (77.46)	15 (21.13)	1 (1.41)	0 (0.00)			
Intermediate	97 (43.50)	62 (63.92)	31 (31.96)	4 (4.12)	0 (0.00)			
Low profile	36 (16.14)	18 (50.00)	12 (33.33)	6 (16.67)	0 (0.00)			
Class						9.16	0.01	0.203
Class 1	118 (52.91)	70 (59.32)	40 (33.90)	8 (6.78)	0 (0.00)			
Class 2	105 (47.09)	82 (78.10)	20 (19.05)	3 (2.86)	0 (0.00)			

### 3.4 Influencing factors of learning outcomes

The extent of students’ pre-class preparation, including careful collection, analysis, and organization of materials, significantly influenced learning outcomes (*p* < 0.05, |*φ*| = 0.219). Among the 44.39% of students who thoroughly prepared, 96.97% met or exceeded their expected learning objectives. Additionally, 9.87% of the students had an average level of preparedness, although 77.27% of them still met or exceeded the expected learning outcomes. A significant correlation was also observed between the time spent on pre-class preparation and learning effectiveness (*p* < 0.05, |*φ*| = 0.196). Of the students who devoted fewer than 5 h to preparation, 20% did not meet their learning objectives, compared to only 5.18% of those who prepared for more than 5 h.

Active participation in offline PBL sessions was reported by 40.36% of students, among whom 95.56% met or surpassed their expected learning objectives. Among the 59.64% of students who reported lower levels of participation, 90.98% still met or exceeded their learning goals. However, no statistically significant difference was found between active and less active students in terms of achieving their learning objectives (*p* = 0.194, |*φ*| = 0.087) (see Table 3).

**Table 3:** Analysis of factors influencing learning outcomes (%).

Influence factors	Cases (*n* = 223)	Learning effect		χ2	*p*	*|φ|*
		Not meeting expectations (*n* = 16)	Meet or exceed expectations (*n* = 207)			
Carefully prepare study materials before class				10.705	0.013	0.219
That’s true	99 (44.39)	3 (3.30)	96 (96.97)			
Basically true	101 (45.29)	8 (7.92)	93 (92.08)			
General	22 (9.87)	5 (22.72)	17 (77.27)			
Not very prepared or occasionally prepared	1 (0.45)	0 (0.00)	1 (100.00)			
Not prepared at all	0 (0.00)	0 (0.00)	0 (0.00)			
Adequate level of preparation (preparation time before class ≥5 h or <5 h)				8.561	0.003	0.196
≥5 h	193 (86.55)	10 (5.18)	183 (94.82)			
<5 h	30 (13.45)	6 (20.00)	24 (80.00)			
The level of enthusiasm for participation (self-evaluation)				1.689	0.194	0.087
Very or quite enthusiastic	90 (40.36)	4 (4.44)	86 (95.56)			
Medium or low profile	133 (59.64)	12 (9.02)	121 (90.98)			

## 4 Discussion

### 4.1 The modified blended PBL teaching model is well received by students

Nursing Care for Women with Hypertensive Disorders of Pregnancy was selected as the topic for this study, which implemented PBL approach combining both online and offline instruction. The study assessed students’ acceptance of this teaching model, their overall evaluation, and their satisfaction with the learning outcomes. The majority of participants were familiar with the PBL pedagogy and generally considered it superior to traditional teaching methods, reporting that the learning outcomes met or even exceeded their expectations. Previous research also supports the effectiveness of PBL from both scientific and practical perspectives (15), indicating that the PBL approach offers distinct advantages and potential in Obstetrics and Gynecology Nursing education. In this study, 95.07% of students expressed satisfaction with the course, and 99.1% were satisfied with the teacher, 92.82% of students expressed greater satisfaction with this blended method compared to traditional teaching approaches.

In the overall evaluation of the course, a certain proportion of students expressed an “unsure” attitude, suggesting that they may not have had a thorough understanding of the teaching model prior to the course. Therefore, the teaching team could provide a detailed introduction to the teaching model before the course begins, to enhance student engagement and improve teaching satisfaction.

### 4.2 The impact of classroom participation on teaching evaluation

Students’ classroom participation significantly influenced their overall course evaluation when rating the course as “excellent,” “good,” “medium,” and “poor.” Students who actively participate in classes tend to rate the course more favorably, indicating a positive correlation between student participation and course evaluation ratings. In this study, among the 8.52% of students who demonstrated highly active participation, 89.47% rated the course as “excellent.” Similarly, among the 31.84% of students who showed relatively active participation, 77.46% gave an “excellent” rating to the course. Classroom participation is widely recognized as an important indicator of course evaluation (16, 17). Students who participated more actively tended to assign higher ratings to the course. The results indicate a statistically significant association between students’ level of active participation in class and their course evaluations (*p* = 0.002, |*φ*| = 0.309), which represents a medium effect. This suggests that students’ classroom participation is an important factor influencing their course evaluations. A significant difference was observed in course evaluations between the two classes (*p* = 0.01, |*φ*| = 0.203). Although the two classes differed slightly in their evaluations of the course, the small effect size indicates that this difference may not be substantial enough to warrant major decision making changes.

Classroom participation has been conceptualized across three dimensions: behavioral, cognitive, and emotional engagement (18). At the classroom level, teaching quality has been identified as a predictor of behavioral engagement (19), and classes with higher overall behavioral engagement were associated with better academic performance. At the individual level, behavioral engagement was correlated with achievement (20).

Students who actively participate in class exhibit a positive academic emotional state. Fostering positive academic emotions is essential in nursing education, as it can enhance SRL abilities and promote lifelong learning (21). SRL serves as a mediator between perceptions of the medical education environment and learning engagement. Therefore, nursing educators should strive to optimize the online learning environment through measures such as designing intelligent teaching platforms, adopting interactive pedagogical methods, reinforcing professional values, and encouraging peer learning, all of which can support student engagement in the new medical education context (22).

The teaching model employed in this study effectively stimulated students’ enthusiasm for participation, established online and offline platforms for full teacher and student interaction, enhanced students’ sense of academic achievement, and thereby improved teaching satisfaction. Thus, it is essential to integrate student participation, course satisfaction, and teaching quality into a cohesive framework (23). Participation in offline classes primarily entails behavioral engagement, which may directly shape students’ overall perception of the course. However, certain biases, such as self report bias and social desirability bias, may have influenced the results to some extent. For instance, when completing the survey, students might not have selected options based on objective reality due to the influence of social desirability concerns.

### 4.3 Classroom participation did not affect the learning outcomes

No significant association was observed between in class participation and achieving expected learning outcomes (*p* = 0.194, |*φ*| = 0.087), indicating that participation may be less critical than preparation or other variables in this context. Notably, active participation in class did not significantly influence students’ learning outcomes, even those with lower participation levels reported meeting their own learning expectations. It is generally accepted that low participation is associated with lower teaching quality (24), and that classroom activity is positively correlated with evaluations of teaching effectiveness (25). However, the findings of this study confirm that class participation level does not always correlate positively with learning outcomes. While highly engaged students achieved the expected learning results, those with lower participation levels also met or even exceeded expectations. Among the 40.36% of students who were highly active in class, 95.56% achieved or exceeded the expected learning outcomes. Similarly, among the 59.64% of less active students, 90.98% still achieved or exceeded the expected results. Students may achieve their learning goals through various channels, such as online self study, group discussions, and in class reflection, rather than relying exclusively on active participation during lessons.

A comparative study found that the PBL group out performed the traditional teaching group in four areas: breadth of knowledge, learning interest, learning efficiency, and collaborative skills, without emphasis on classroom participation (26). This indicates that students can attain desired learning outcomes through alternative learning activities. Research also reveals that self assessment enhances self awareness, goal orientation, and task planning among medical students in PBL tutorials. Furthermore, external feedback and opportunities for revision improved students’ self regulation during learning (27).

The PBL model fosters the development of students’ SRL abilities, enabling them to evaluate their learning progress and adjust their strategies accordingly. Therefore, PBL instruction should adhere to a student-centered approach (28), accommodate diverse learning needs, and encourage participation in ways aligned with individual learning preferences. In addition, the evaluation of teaching effectiveness should be comprehensive, incorporating not only in class performance but also out of class engagement and other learning behaviors.

### 4.4 Impact of pre-class preparation on teaching effectiveness

The results indicate a significant positive association between adequate pre-class preparation and expected learning outcomes (*p* = 0.013, |*φ*| = 0.219, weak to moderate effect), though other factors also contribute to learning effectiveness. A significant but weak relationship was found between preparation time and learning outcomes (*p* = 0.003, |*φ*| = 0.196), suggesting preparation duration is one of several influencing factors. Students’ evaluation of “whether the learning outcome met expectations” served as one of the primary indicators for assessing the teaching effectiveness of the course. It was found that thorough preparation before class significantly enhanced learning outcomes, pre-class preparation time is a predictor of learning outcomes, but not the only factor. Preparation efficiency also correlates with in class learning effectiveness. Among the 86.55% of students who spent ≥5 h on pre-class preparation, 94.82% achieved the expected learning outcomes. Among the 13.45% of students who spent <5 h on pre-class preparation, 80.00% still achieved the expected learning outcomes. Previous experiments have demonstrated that adequate pre-class preparation and discussion are important factors in improving teaching effectiveness (29), which is consistent with the results of this study.

This study evaluated whether students carefully studied the online materials and whether their online study time reached or exceeded 5 h. The results indicate that students who actively collected, analyzed, and organized learning materials before class generally rated the learning effectiveness of the course more highly. This positive effect was particularly pronounced when online study time was 5 h or more. Similar findings have been reported in earlier studies, which showed that organizing students into online groups, clarifying learning tasks and objectives, and having them complete pre-class experiments effectively improved the quality of the students’ lab reports (28).

Therefore, sufficient preparation prior to offline classes is essential for achieving optimal learning outcomes in a PBL environment. Teachers can provide a wide range of resources for students to utilize during pre-class guidance, and a PBL problem-oriented learning approach can enhance students’ pre-class preparation efficiency.

## 5 Research applications and limitations

The modified blended PBL approach introduced in this study can be applied to other nursing courses, achieving a high level of integration between online and offline instruction based on PBL methodology. It makes full use of online resources to support students’ SRL skill and enhances their ability to manage independent learning. The student-centered offline teaching model effectively stimulates learning motivation, fosters a sense of achievement, and promotes professional identity formation. Teachers play a facilitative role by providing guidance and feedback, helping students consolidate knowledge, and offering opportunities to develop critical thinking skills. This model is suitable for medical and nursing students and facilitates the transformation and application of theoretical knowledge. In the future, the evaluation dimensions of teaching quality could be expanded, and instructors could become more involved in online learning activities. Additionally, teachers should pay greater attention to individual differences among students in PBL settings.

This study has several limitations. First, it did not investigate the reasons for divergent PBL teaching evaluation outcomes or explore the influencing factors and improvement strategies for learning outcomes in Obstetrics and Gynecology Nursing education. Additionally, the lack of baseline assessments, such as prior academic performance, student motivation, or instructional differences, which limits the generalizability of the findings, as these variables may affect learning outcomes. Other limitations include the short term evaluation period without longitudinal follow up, potential variability in PBL implementation across instructors, and self selection bias in online preparatory activities.

Employing a cross sectional descriptive design without a control group also restricted the ability to control for confounding variables. Future research should incorporate control groups and pre-class assessments (e.g., students’ academic background and instructional competence) to more rigorously evaluate the teaching model’s effectiveness. Further recommendations include using validated instruments to measure satisfaction and learning outcomes, conducting longitudinal studies to assess knowledge retention, and integrating qualitative methods such as interviews to gain deeper insights into the experiences of students and teachers.

## 6 Conclusion

Existing research on Modified blended PBL has largely emphasized knowledge acquisition, satisfaction, and engagement. This study innovatively evaluates the impact of online self-directed learning, pre-class preparation, and in-class participation.

Results indicate that pre-class preparation significantly enhances learning outcomes, underscoring the need to provide structured resources to foster self-directed learning. While class participation boosts teaching evaluations, as active contributors gain more value from peer and instructor interaction, it does not directly correlate with learning outcomes. Nonetheless, cultivating an engaging classroom atmosphere remains crucial for overall satisfaction.

The PBL model effectively promotes students’ SRL ability, enabling students to assess and adapt their approaches based on comprehension. Thus, lower participation does not imply poor knowledge acquisition. This highlights a core strength of blended PBL: supporting self management skills without compromising academic effectiveness, solidifying its relevance in contemporary nursing education. Based on this study’s findings, here are concise recommendations for PBL teaching, Focus on pre-class preparation, provide structured resources to guide self-directed learning before class, as this directly improves outcomes. Value quality of participation, foster an engaging atmosphere but recognize that active speaking boosts course satisfaction more than directly impacting test scores. Promote SRL, empower students to manage their own learning process, which is a key benefit of the blended PBL model.

## Data Availability

The datasets presented in this study can be found in online repositories. The names of the repository/repositories and accession number(s) can be found in the article/supplementary material.
